# Suspected clinical chorioamnionitis with peak intrapartum temperature <38^0^C: the prevalence of confirmed chorioamnionitis and short term neonatal outcome

**DOI:** 10.1186/s12887-022-03239-9

**Published:** 2022-04-11

**Authors:** Anvar Paraparambil Vellamgot, Khalil Salameh, Lina Hussain M. Habboub, Rajesh Pattuvalappil, Naser Abulgasim Elkabir, Yousra Shehada Siam, Hakam Khatib

**Affiliations:** 1 Department of Neonatology, Al Wakra Hospital, Hamad Medical Corporation, Al Wakra, Qatar; 2 Department of Obstetrics and Gynecology, Al Wakra Hospital, Hamad Medical Corporation, Al Wakra, Qatar

**Keywords:** Chorioamnionitis, Peak intrapartum temperature, Antipyretics

## Abstract

**Background:**

Chorioamnionitis (CA) affects up to 3.9% of all deliveries worldwide and is one of the leading causes of early-onset neonatal sepsis. Fever≥38^0^C is an essential criterion for the diagnosis of clinical CA. Obstetricians frequently take the maternal risk factors into consideration, and many mothers are treated as CA even with peak intrapartum temperature (PIT) between 37.6^0^C to 37.9^0^C if they have other clinical signs and risk factors.

**Aim:**

To estimate the prevalence of confirmed chorioamnionitis and adverse neonatal outcomes among those mothers with PIT below 38^0^C.

**Materials and methods:**

Retrospective chart review among mothers delivered at Al-Wakra Hospital, Qatar, between1^st^January2016 to 31^st^December 2019 with a clinical suspicion of CA.

**Results:**

Among 21,471 mothers, 442 were suspected of having CA (2.06%, 95% CI 1.88 to 2.26%). After exclusions, 415 were included in the study, 203(48.9%) mothers had PIT between 37.6-37.9^0^C. There was no significant difference in the rate of confirmed CA between the low (<38^0^C) and higher (≥38^0^C) temperature groups (25.4%Vs.31.3%, OR0.75, 95%CI0.46-1.25 , p.262). More patients in the low-temperature group received paracetamol for PIT between 37.6 to 37.9 ^0^C, while it was less frequently used for such milder elevation in higher temperature group (88.2%Vs.38.9%, OR11.69, 95% CI 6.46-2.15, *p* <.001).

**Conclusion:**

The incidence of suspected clinical CA in our institution was within the international rates. Although nearly half of the mothers with suspected clinical CA had peak temperature below the recommended diagnostic criteria, the rate of confirmed CA and neonatal outcome was not significantly different from those with PIT≥38^0^C. Early antipyretic use might have affected further elevation of temperature.

**Supplementary Information:**

The online version contains supplementary material available at 10.1186/s12887-022-03239-9.

## Introduction

Intraamniotic infection (IAI) or Chorioamnionitis (CA)refers to the infection of the amniotic fluid, membranes, placenta, and/or deciduas. At term, CA complicates approximately 1 to 4 percent of deliveries overall [1–3]. CA is a well-recognized risk factor for early-onset neonatal sepsis (EOS) [4, 5].

Overall, 40 percent of cases of EOS have associated CA [2]. Adverse fetal/neonatal outcomes of CA include perinatal death, asphyxia, early-onset neonatal sepsis, septic shock, pneumonia, meningitis, intraventricular hemorrhage, cerebral white matter damage, and long-term disability, including cerebral palsy, as well as morbidity related to preterm birth [5–10].

The acute symptomatic condition which often presents with fever, uterine tenderness, genital discharge, tachycardia, and evidence of fetal infection, is termed clinical chorioamnionitis while the finding of acute placental inflammatory changes with or without evidence of infection is termed histological chorioamnionitis [11].

Among very preterm infants (< 32 weeks) born spontaneously, the risk of cerebral palsy at a corrected age of 2 years was higher among those exposed to clinical chorioamnionitis but not histologic chorioamnionitis [12]. Among term neonates with perinatal asphyxia, both clinical and histological chorioamnionitis were associated with a persistent state of metabolic acidosis in neonates which may contribute to adverse neurologic outcome [13].

Prolonged labor and premature rupture of membranes, multiple digital vaginal examinations, cervical insufficiency, nulliparity, meconium-stained amniotic fluid, internal fetal or uterine contraction monitoring, and presence of genital tract pathogens are important risk factors for CA [1, 2].

The manifestations include fever, leukocytosis >15000 cells/mm^3^, feto-maternal tachycardia, uterine tenderness, foul-smelling amniotic fluid, and sometimes bacteremia.

Histopathology of affected placentae may reveal chorioamnionitis with or without funisitis. The maternal complications include dysfunctional labor, localized infections, and sepsis.

The conventional diagnostic criteria for CA clinical diagnosis included Peak Intrapartum Temperature (PIT) ≥38^0^C plus at least one feature from leukocytosis>15000 cells/mm^3^, fetal tachycardia >160 beats per minute, maternal tachycardia >100 beats per minute, uterine tenderness, and foul-smelling amniotic fluid.

The recent diagnostic criteria suggested by a National Institute of Child Health and Human Development Workshop expert panel and endorsed by the American College of Obstetricians and Gynecologists (ACOG) [14] is as follows:

A presumptive diagnosis of CA can be made in women with fever (≥39.0°C [102.2°F] or 38.0°C [100.4°F] to 38.9°C [102.02°F] on two occasions, 30 minutes apart) without another clear source plus one or more of the following: a)baseline fetal heart rate >160 beats/min for ≥10 minutes, excluding accelerations, decelerations, and periods of marked variability, c)maternal white cell count >15,000 cells/mm^3^ b)purulent-appearing fluid coming from the cervical os, visualized by speculum examination. The new criteria excluded maternal tachycardia and uterine tenderness.

A confirmed CA is based on the presence of the above criteria plus positive amniotic fluid test result (gram stain, glucose level, or culture results consistent with infection) or placental pathology demonstrating histologic evidence of placental infection or inflammation [14].

Women with clinical CA should receive broad-spectrum antibiotics promptly to initiate treatment of both the mother and fetus [14]. Maternal intrapartum fever has been associated with a higher frequency of fetal tachycardia, intervention for non-reassuring electronic fetal monitoring, operative vaginal delivery, cesarean delivery, neonatal depression, neonatal encephalopathy, perinatal arterial ischemic stroke, neonatal seizures, and NICU admission [15–33]. Hence fever should be treated by antipyretics.

The American Academy of Pediatrics Committee on Fetus and Newborn (AAP-COFN) recommended partial sepsis workup and presumptive antibiotics to all newborn babies born with suspected maternal CA [34]. Current evidence-based strategies like Early-onset Sepsis risk Calculator (EOSCAL) [35] focuses on maternal temperature and associated risk factors rather than the clinical diagnosis of chorioamnionitis.

Although fever (≥38^0^C) is an essential criterion for diagnosing CA, obstetricians frequently take the risk factors into consideration, and many mothers are treated as CA even with PIT 37.6-37.9^0^C if they have other clinical signs or risk factors. An audit conducted in AWH (2018) showed that 50% of clinically suspected cases of CA did not satisfy the diagnostic criteria, including fever. We observed that most mothers received antipyretics at a lower temperature range (37.6 to 37.9^0^C). We hypothesized that early use of antipyretics might be blunting the clinical picture of CA.

## Objectives

### Primary objective

To estimate the prevalence of confirmed CA among mothers who delivered in AWH between Jan 2016 and Dec 2019 and had suspected clinical CA with a PIT between 37.6^0^C -37.9^0^C.

### Secondary objectives

1. To estimate the incidence and trend of suspected clinical CA among mothers delivering at AWH.
2. To compare the maternal risk factors, clinical features, final diagnosis, and short-term neonatal outcome between those with PIT <38^0^C (group 1) and those with temp 38^0^C and more (group 2).

## Methods

### Study design

Retrospective data analysis.

### Setting

The study was conducted at Al Wakra Hospital (AWH), Qatar, from July to Dec 2020. This was a retrospective data analysis. Data was collected for neonates exposed to suspected clinical chorioamnionitis and admitted to the NICU between 1^st^ January 2016 and 31^st^ December 2019.

### Participants

#### Inclusion criteria

All the chorioamnionitis-exposed neonates born during the study period and their mothers were included. A case was considered as suspected clinical chorioamnionitis if the obstetrician strongly considered the diagnosis of clinical chorioamnionitis based on clinical features and risk factors. For comparison, subjects were divided into 2 groups (Group 1 = PIT 37.6-37.9^0^C, Group 2 = PIT ≥38^0^C).

### Exclusion criteria

- Cases of CA resulting in abortions or intrauterine fetal deaths
- Mothers with a known focus of infection other than CA
- Patients transferred in from the other facilities
- Babies with significant congenital malformations,
- Incompletely documented files
- Mothers with documented intrapartum temperature <37.6^0^C

### Variables

#### Maternal data

1. Basic data
  Age, parity, gravida, gestational age at the time of delivery, date and time of birth, and type of delivery were collected.
2. Risk factors
  These included Group B streptococcus (GBS) colonization status**,** artificial or spontaneous rupture of membranes (AROM or SROM), meconium-stained amniotic fluid (MSAF), induced or spontaneous labor, rupture of the membranes (ROM) to delivery duration, number of vaginal examinations during labor, and whether received epidural analgesia or not. The length of labor was calculated as the duration in hours from the onset of regular contractions (2 to 3 moderate to strong contractions/10minute) to the delivery of the baby.
3. Data about diagnostic criteria and intrapartum management
  The data included PIT during labor**,** use of antipyretic for temperature below 38^0^C, fetal tachycardia and maternal tachycardia**.** For those with peak temperature 38^0^C or more, previous temperature recordings were reviewed to find if there were any readings between 37.6^0^C and 37.9^0^C for which antipyretics were given. Data regarding uterine tenderness, foul-smelling fluid, and purulent cervical discharge were not well- documented and hence were not collected. Data regarding intrapartum GBS prophylaxis and broad-spectrum antibiotic use were collected with the timing in relation to the delivery. White blood cell count (WBC) before delivery, highest C-reactive protein before delivery, blood culture, and mode of delivery were checked.
4. Data about placental pathology and culture
  As both placental histopathology and amniotic fluid culture have limited utility in immediate perinatal patient care, these were not routinely performed in our practice. Placental histopathology was performed only among a small proportion of the study sample. Because of the limited utility and invasive nature, amniotic fluid aspiration was not performed in any of the subjects. Our obstetric team considered placental culture as a surrogate for amniotic fluid culture. Patients with either positive histopathology or placental culture were considered to have a confirmed CA. The growth of any significant pathogenic bacteria from the placenta was included.

### Neonatal data

1. Basic data
  Date and time of birth**,** gestational age at birth**,** birth weight**,** and sex.
2. Perinatal data
  Apgar score**,** need for positive pressure ventilation at birth, and temperature at birth.
3. Details regarding diagnosis, management, and outcome
  These included symptoms upon admission WBC count**,** highest CRP during the first 24 hours, blood culture, cerebrospinal fluid study, highest respiratory support needed, antibiotic duration, discharge diagnosis, length of stay in NICU and readmission for sepsis within one week after discharge.

### Data sources

Since all the babies exposed to suspected clinical CA were admitted to the newborn nursery, the NICU admission registry was reviewed to identify the cases. Additional data about the total deliveries, including the intrauterine fetal deaths, were obtained from the labor room registry and the medical records department. The maternal and neonatal variables were collected from the electronic documentation software ‘Cerner Millennium.'

### Statistical analysis

All Statistical analyses were done using statistical packages SPSS 22.0 (SPSS Inc. Chicago, IL). Descriptive statistics were used to summarize the baseline data. The results were reported as mean and standard deviation (SD) or frequencies and percentages as per the type of the data. Associations between two or more categorical variables were assessed using the chi-square (χ2) test or Fisher Exact test wherever appropriate. Quantitative variables means between the two and more than two independent groups were analyzed using unpaired t-test or one-way analysis of variance (ANOVA). All *P* values presented are two-tailed, and *P*-value <0.05 was considered statistically significant.

### Missing data

Patients with missing variables were excluded from the study.

### Consent and ethical considerations

This was a retrospective chart review. All the subjects were de-identified by coding. Hence the Institutional Review Board (IRB) of Hamad Medical Corporation waived off the need for consent from the subjects.

## Results

**Fig 1.**
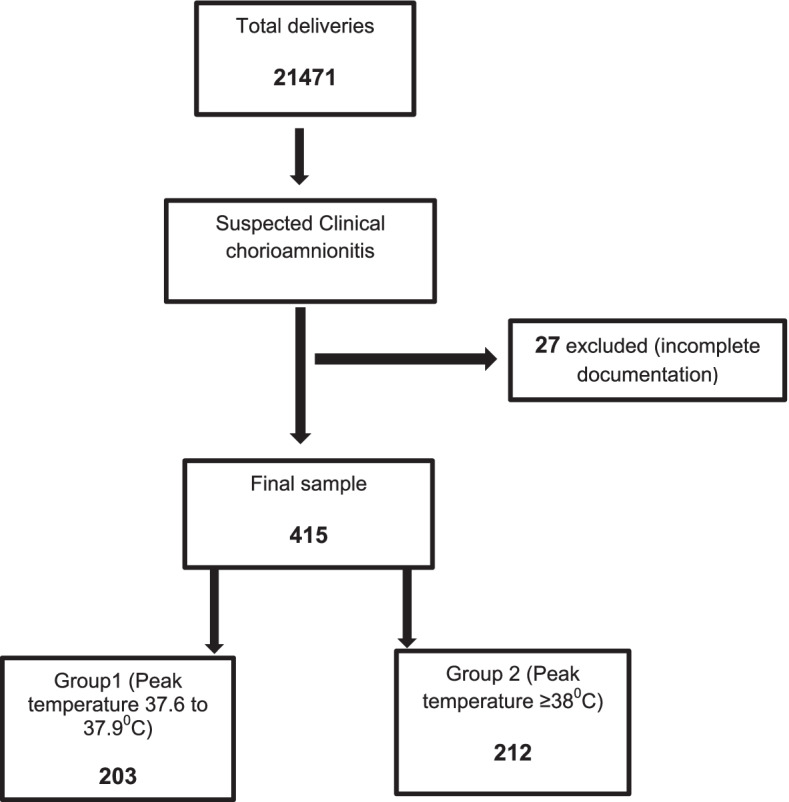
Sample acquisition

Between Jan 2016 and Dec 2019 (48 months), AWH recorded 21554 deliveries (Table 1). Eighty-three cases of intrauterine fetal deaths were excluded. All cases of abortions were also excluded. The final number of deliveries included was 21471. Among them, 442 (2.06%, 95% CI 1.88 to 2.26 %) mothers were suspected of having clinical chorioamnionitis. Twenty-seven cases were excluded because of incompletely documented data. The final study population included 415 mother-infant pairs (Fig. 1). There was a steady increase in the incidence of the suspected cases from 1.16% in 2016 to 3.09% in 2019, with the rate reaching more than double in the 4^th^ year.

**Table 1:** Incidence of suspected clinical chorioamnionitis

**Year**	**Total mothers delivered** **(Excluding IUFD and abortions)**	**Suspected clinical chorioamnionitis** *N*(% of total deliveries)	**Cases included for the study**
2016	5223	61 (1.16%)	58
2017	5771	113 (1.96%)	107
2018	5633	118 (2.09%)	110
2019	4844	150 (3.09%)	140
Total	21471	442 (2.06%)	415

*IUFD* Intrauterine fetal death

The mean maternal age and gestational ages were 28.4 years and 39 weeks, respectively (Table 2). 6.3% of the deliveries were before 37 weeks gestation. 203 (48.9%) of the mothers with suspected clinical CA had their PIT between 37.6^0^C to 37.9 ^0^C (Table 1). Placental histopathology or placental culture was performed for 138 mothers in this low-temperature group, and 25.4% were confirmed to have CA ( 95% CI 18.8% to 33.2%). Among 212 mothers with PIT ≥ 38^0^C, at least one confirmatory test was done for 140 patients, and 31.3% had a confirmed diagnosis (Table 3). There was no statistically significant difference in the rate of confirmed CA between the low and high-temperature groups (*p* = .262).

**Table 2:** Baseline data

**Total *N* = 415**		
Mean maternal age (SD)		28.4 (4.57)
Nulliparity *N* (%)		298 (71.8%)
Mean Gestational age in weeks (SD)		39 (1.8)
Premature <37 weeks *N* (%)		26(6.3%)
PIT 37.6^0^C to 37.9^0^C *N* (%)		203 (48.9%)
Peak intrapartum temperature 38^0^C or more *N* (%)		212 (51.1%)
Mean birth weight in gm (SD)		3312(524)
Sex-newborn *N* (%)	Male	215(51.8%)
	Female	200(48.2%)

*N* Number*, SD* Standard deviation

**Table 3:** Comparison-maternal risk factors

				**Comparison -Group (1) Vs. Group (2)**		
	**Total patients *N* =415**	**Group (1) PIT 37.6 to 37.9**^**0**^**C *N* = 203**	**Group (2) PIT 38**^**0**^**C or more *N*=212**	**Mean difference (95% CI)**	**Odds ratio (95% CI)**	***P*-value**
Mean Maternal age in years (SD)	28.42(4.57)	28.16 (4.63)	28.67(4.5)	-0.503 (-1.38 to 0.38)		0.263
Mean Gestational age in weeks (SD)	38.99(1.82)	38.8 (2.06)	39.13(1.54)	-0.285 (-0.64 to 0.65)		0.113
Gestational age <37 weeks N (%)	26 (6.3%)	17 (8.4%)	9 (4.2%)	2.06 (0.89 to 4.74)		0.083
Nulliparity *N* (%)	298 (71.8%)	156(76.8%)	142 (67%)		1.64 (1.06 to 2.53)	0.026
GBS carrier *N* (%)	36/174 (20.7%)	16/86 (18.6%)	20/88 (22.7%)		0.78 (0.37 to 1.62)	0.502
Epidural analgesia *N* (%)	343(82.7%)	172 (84.7%)	171 (81%)		1.29 (0.78 to 2.17)	0.320
SROM *N* (%)	158(38.1%)	85(41.9%)	73 (34.4%)		1.37 (0.92 to 2.04)	0.119
Mean ROM duration in hours (SD)	18.18 (24.2)	21.51(28.65)	15(18.5)	6.51 (1.83 to 11.18)		0.007
ROM 18 hrs or more *N* (%)	131(31.6%)	74 (36.5%)	57(26.9%)		1.56 (1.03 -2.57)	0.036
Induction *N* (%)	149(35.9%)	77 (37.9%)	72(34%)		1.19 (0.79 to 1.78)	0.400
Regular contractions at the time of admission *N* (%)	144(34.7%)	62(30.5%)	82(38.7%)		0.69 (0.46 to 1.05)	0.082
Mean duration of labor (SD) in hours	13.44(8.49)	13.53(8.08)	13.34(8.91)	0.186 (-1.46 to 1.84)		0.824
Duration of labor>15 hours *N* (%)	143(33.7%)	72 (35.5%)	68 (32.1%)		1.16 (0.78 to 1.75)	0.465
Mean no of vaginal examinations (SD)	5.79(2.42)	5.97(2.44)	5.63(2.4)	0.338 (-0.129to0.80)		0.155
8 or more vaginal examinations *N* (%)	88(21.4%)	48 (23.6%)	40(18.9%)		1.33 (0.83 to 2.14)	0.234
MSAF N (%)	105(25.3%)	50 (24.6%)	55(25.9%)		0.93 (0.59 to 1.45)	0.758

*PIT* Peak intrapartum temperature*, N* Number*, SD* Standard deviation, *CI* Confidence interval, *GBS* Group-B streptococcus, *SROM* Spontaneous rupture of membranes, *ROM* Rupture of membranes, *MSAF* Meconium-stained amniotic fluid

Further, we compared the background risk factors, clinical and laboratory parameters, and neonatal outcomes of the low and high-temperature groups (group 1and group 2). The maternal infection risk factors were compared between the two groups (Table 3).

There was no statistically significant difference in the mean maternal age, mean gestational age, prematurity, GBS carrier state, epidural analgesia, spontaneous rupture of membranes, need for induced labor, mean length of labor, the number of vaginal examinations, and meconium-stained amniotic fluid. Patients in the group1 were significantly more likely to be nulliparous (p .026, OR 1.64, 95% CI 1.06 to 2.53) and had a higher rate of prolonged rupture of membranes more than 18 hours (*p* .036, OR 1.56, 95% CI 1.03 to 2.57).

In Table 4, we compared the maternal clinical characteristics, lab findings, and the treatments received. Those in the higher temperature group were more likely to have maternal and fetal tachycardia (*p* .010 and .020, respectively).

**Table 4:** Comparison of maternal clinical and lab findings, Antipyretic use, mode of delivery, and prevalence of confirmed chorioamnionitis

				**Comparison -Group (1) Vs. Group (2)**	
	**Total patients** *N*=415	**Group (1)** **PIT 37.6 to 37.9**^**0**^**C** *N*= 203	**Group (2)** **PIT 38**^**0**^**C or more** *N*=212	**Odds ratio** **(95% CI)**	*P *value
Maternal tachycardia	251 (60.5%)	110(54.2%)	141 (66.5%)	0.59 (0.40 to 0.89)	0.010
Fetal tachycardia	198 (47.7%)	85(41.9%)	113(53.3%)	0.63 (0.43 to 0.93)	0.020
WBC >15 *N* (%)	188 (45.3%)	90(44.3%)	98(46.2%)	0.93 (6.23 -1.36)	0.699
CRP>20 *N* (%)	213/329 (64.7%)	97/152 (63.8%)	116/177 (65.5%)	0.93 (0.59 to 1.46)	0.745
Positive blood culture(mother)*N* (%)	25/208 (12%)	6/68 (8.8%)	19/140 (13.6%)	0.62 (0.23 to 1.62)	0.323
Intrapartum antibiotics at least 2 or more hours before delivery	233 (56.1%)	119 (58.6%)	114 (53.8%)	1.22 (0.83 to 1.79)	0.320
Received paracetamol for temperature below 38^0^C *N* (%)	215/297 (72.4%)	179/203(88.2 %)	37/95 (38.9%)	11.69 (6.46 to 21.15)	<0.001
LSCS	190 (45.8%)	91 (44.8%)	99(46.7%)	0.93 (0.64 to 1.37)	0.706
Instrumental vaginal delivery *N* (%)	97 (23.4%)	49(24.1%)	48(22.6%)	1.09 (0.69 to 1.71)	0.719
Positive Placenta culture *N* (%)	62/275 (22.5%)	25/134(18.7%)	37/141(26.2%)	0.65 (0.36 to 1.15)	0.132
Positive placental pathology *N* (%)	29/45 (64.4%)	12/20 (60%)	17/25 (68%)	0.71 (0.21 to 2.41)	0.577
Confirmed chorioamnionitis (based on placental culture or histopathology) *N* (%)	82/288 (28.5%)	35/138 (25.4%)	47/140 (31.3%)	0.75 (0.46 to 1.25)	0.262
Confirmed chorioamnionitis (based on the placental culture of LSCS cases or by histopathology) *N* (%)	53/177 (29.9%)	19/87 (21.8%)	34/90 (37.8 %)	0.46 (0.24 to 0.89)	0.021

*PIT* Peak intrapartum temperature*, N* Number*, CI* Confidence interval, *WBC* White blood cells, *CRP* C-reactive protein, *LSCS* Lower segment caesarian section

There were no significant differences in the prevalence of maternal CRP>10 mg%, WBC >15 x10^3^, positive blood Cultures, intrapartum antibiotic use, and mode of delivery.

In the low-temperature group, 88% received paracetamol soon after the spike. In the higher temperature group, 95 babies (44.8%) had at least one preceding temperature reading between 37.6 and 37.9^0^C, and only 38 % of them received paracetamol for this mild elevation of temperature. Hence, when compared to group 2, mothers in group 1 were significantly more likely to receive paracetamol for the borderline elevation of temperature (*p* <.001, OR 11.69, 95% CI 6.46 to 21.15).

Placental histopathology was performed in 45 patients only. CA was seen in 29 placentae (64%). Among them, 8 had severe CA, and 6 had funisitis. However, there was no statistically significant difference between positive placental pathology between the two groups (*p* .577, OR 0.71, 95% CI 0.21 to 2.41).

Placenta culture was performed in 275 mothers and was positive in 22.5%. The organisms isolated were as follows:

There were 14 different bacteria cultured from 63 placentae. GBS and E. Coli formed nearly two-thirds of all organisms cultured (Table 5).

**Table 5:** Organisms cultured from the placentae

**Organism cultured**	**Number ( %)**
Group B Streptococcus	29 (44.6%)
Escherichia Coli	11 (16.9%)
Bacteroides sp.	4
Streptococcus anginosus	4
Klebsiella sp.	3
Coagulase negative Staphylocccus aureus	3
Beta hemolytic streptococci	2
Enterococcus fecalis	2
Methicilin resistant Staph aureus ( MRSA)	2
Streptococcus bovis	1
Peptostreptococcus	1
Hemophilus Influenza	1
Coliform sp.	1
Streptococcus perfringens	1
Total	65

When patients with positive placental culture or histopathology were assumed as confirmed cases, 25.4 % of patients in the low PIT group and 31.3% in the higher PIT group had CA. The difference was not statistically significant (*p* = .262).

The placenta in a vaginal delivery is very likely to be contaminated with vaginal flora. After excluding cultures done from the vaginally delivered placenta, 21 % of patients in group 1 had a confirmed diagnosis of chorioamnionitis. Although the number is significantly less than that of group 2, it is clinically significant.

The short-term outcome of the neonates is compared in Table 6. During the study period, all the babies born to mothers with suspected clinical CA were admitted to NICU and received IV antibiotics for at least 48 hours. Babies in group 1 were more likely to be symptomatic (*p*.016, OR 1.8, 95%CI 1.81 to 2.92). Respiratory distress was present among all the babies who were symptomatic. Four babies had positive blood CS, and all of them were from group 2. However, the combined outcome of sepsis (both culture-positive and culture-negative) and congenital pneumonia was similar between the two groups (7.4 % in group 1 Vs. 9% in group 2, *p* 0.341). There was no statistically significant difference in the need for any resuscitation at birth, need for respiratory support in NICU, mean white cell count, CRP, or length of stay. In group 1, five babies required mechanical ventilation and only one baby required ventilation in group 2.

**Table 6:** Short term outcomes of the neonates

					**Comparison -group (1) Vs. group (2)**	
	**Total patients** *N* =415	**Group 1** **PIT 37.6 to 37.9**^**0**^**C** *N*=203	**Group 2** **PIT 38**^**0**^**C or more** *N*=212	**Mean difference** **(95% CI)**	**Odds Ratio** **(95% CI)**	*P*-value
Mean temp. at birth	37.53(0.68)	37.44(0.59)	37.62(0.74)	-.18 (-0.38 to -0.49)		0.007
Temp at birth >37.5 *N* (%)	250 (60.2 %)	130 (64%)	120(56.6%)		1.36 (0.92 to 2.03)	0.122
Any resuscitation at birth *N* (%)	28 (6.7%)	14(6.9%)	14 (6.6%)		1.05 (0.69 to 1.50)	0.905
Respiratory distress *N* (%)	86 (20.7%)	52 (25.6%)	34 (16%)		1.80 (1.11 to 2.92)	0.016
Mean white cell count (SD)	17.33(5.42)	17.41 (5.85)	17.25(5.01)	0.16 (-0.89 to 1.20)		0.770
CRP >10 *N* (%)	55/149 (36.9%)	25/81 (30.9%)	30/68 (44.1%)		0.57 (0.29 to 1.10)	0.095
Any respiratory support in NICU *N* (%)	74 (17.8%)	41 (20.2%)	33 (15.6%)		1.37 (0.83 to 2.28)	0.218
Mechanical ventilation *N* (%)	6 (1.4%)	5	1			
Neonatal sepsis or pneumonia *N* (%)	34 (8.2%)	15(7.4%)	19 (9%)		0.81 (0.40 to 1.64)	0.341
Culture proven sepsis *N* (%)	4 (0.96%)	0	4(1.89%)			
NICU stay >3 days	73 (17.6%)	29 (14.3%)	44(20.8%)		0.64 (0.38 t0 1.07)	0.084

*PIT* Peak intrapartum temperature*, N* Number*, CI* Confidence interval, *SD* Standard deviation, *CRP* C-reactive protein, *NICU* Neonatal intensive care unit

## Discussion

We observed a steady increase in the incidence of the suspected clinical CA from 1.16% in 2016 to 3.09% in 2019. The observed incidence is similar to observations in the previous studies. A recent meta-analysis by Woodd et al. [36] observed an overall incidence of 3.9% (95% CI 1.8% to 6.8%). The wide variation is due to several factors, including differences in ascertainment (prospective studies report higher rates than retrospective studies), differences in the prevalence of risk factors in the populations studied, use of different diagnostic criteria (e.g., clinical versus histologic), and temporal changes in obstetric practice [37].

PIT ≥ 38 ^0^C is an essential clinical criterion for the diagnosis of intraamniotic infection [2, 36]. We found that 48.9% of our mothers with suspected clinical chorioamnionitis had their PIT between 37.6^0^C to 37.9 ^0^C.

At least one confirmatory test was done among 138 of this low-temperature group, and 35 (25.4%) of them were positive for CA. This was not much different from the rate of confirmed cases among 140 patients in the high-temperature group (31.3%), and the difference was statistically insignificant (*p* = .262). This finding contradicts the established minimum PIT criteria of 38^0^C. Keeping this in mind, we did further analysis of the baseline risk factors, clinical and laboratory parameters, and neonatal outcome in the two groups.

Prolonged rupture of membranes, prolonged labor, multiple vaginal examinations, nulliparity, meconium staining of amniotic fluid, and GBS carrier state are important risk factors for CA [1, 2, 38–41].

The observed maternal risk factors were similar to the previous studies (Table 7). Similarly, the clinical findings, lab findings, and neonatal outcomes were also comparable to the previously published observations (Table 8). This might justify our clinical suspicion of CA even in the absence of a PIT of 38^0^C and more. In a recent prospective cohort study conducted by Sayyed AA et al. among neonates exposed to suspected CA, 36% had PIT<37.8^0^C [42]. We observed that many of the mothers received intrapartum paracetamol if the temperature increased beyond 37.5^0^C, especially if it happened two or more times. The Obstetric team was concerned about the adverse fetal, maternal, and neonatal outcomes of intrapartum pyrexia and was tempted to use antipyretics at a minimal threshold.

**Table 7:** Maternal risk factors: Comparison to previous studies

**Risk factor**	**Present study:** *N*=415	**Previous studies**
Premature rupture of Membranes	38.1%	Premature rupture occurs in 8% or less of term pregnancy (ACOG 2007 [43]
Rupture of membranes duration	• Mean ROM duration: 18.18 hours • ROM >18 hours: 31.6%	ROM 19.7 hr (Soper et al. 1989 [44]) ROM>18hours has RR 6.9 for IAI (Rickert et al.1998 [45])
Duration of labor labor (Onset of regular contraction to delivery)	Mean duration: 13.44 hours >15 hours: 33.7%	20.9 hours (Soper et al. 1989 [44] Labor duration >15 hours has a RR of 4 for CA (Seaward et al. [38]
Meconium-stained fluid	25.3%	33% (Romero R et al. 1991 [46] 21% (Tran et al. 2003 [39] 11.5% (Venkatesh et al. 2019 [47]
Labor induction	35.9%	45% (Cohill et al. 2012 [48]) 9% (Rikert et al. 1998 [45]) 47.8 % (Venkatesh et al. 2019 [47])
Number of vaginal examinations	Mean no: 5.79	6.1 (Soper et al. 1989 [44])
GBS carrier state	20.7%	18.8% (Venkatesh et al. 2019 [49]) 11% (Shahni et al. 2019 [50])
Nulliparity	71.8%	48% (Cohill et al. 2012 [48]) 70.6% (Venkatesh et al. 2019 [47])

*ACOG* American College of Obstetricians and Gynecologists, *ROM* Rupture of membranes, *CA* Chorioamnionitis, *GBS* Group B streptococcus

**Table 8:** Maternal clinical and lab findings, mode of delivery, and neonatal outcome – Comparison to previous studies

**Parameter**	**Present study** *N*=415	**Previous studies**
Maternal tachycardia	60.5%	20-80 % (Newton et al. 1993 [1])
Fetal tachycardia	47.7%	40-70% (Newton et al.1993 [1])
White blood cells >15	45.3%	70-90 % (Newton et al. 1993 [1])
Positive blood culture for mother	12%	12% (Yoder et al. 1983 [6]) 5-10% (Duff P et al. 2012 [51])
Intrapartum antibiotics	≥ 2 hours before delivery- 56%	>3 hours: 32.2 % (Venkatesh et al. 2019 [47])
Cesarian Section	45.8%	31.3% (Soper et al. 1989 [44]) 36% (Yoder et al. 1983 [6]) 21% (Rikert et al. 1998 [45]) 41.9% (Venkatesh et al. 2019 [47])
Instrumental vaginal delivery	23.4%	4.7% (Venkatesh et al. 2019 [47])
Histologic chorioamnionitis	64.4%	7-85% (Holzman et al. 2007 [52]) 62% (Smullen et al. 1999 [53])
Positive amniotic fluid / placental culture	22.5%	46%- amniotic fluid culture (Romero et al. 2015 [54])
Mean newborn rectal temperature at birth	37.53^0^C	Mean: 37.5 ^0^C (Shalak et al. 2005 [55])
Any resuscitation at birth	6.7%	11.5% (Liebermann et al. 2000 [49])
Neonatal respiratory distress	20.7%%	20% (Yoder et al. 1983 [6]) 24.6% (Venkatesh et al. 2019 [47])
Need for mechanical ventilation	1.4%	0.89% (Venkatesh et al. 2019 [47]) 2.5 % (Rouse et al. 2004 [56])
Culture-proven neonatal sepsis	0.96%	8% (Yoder et al.1983 [6]) 1.3% (Rouse et al. 2004 [56]) 0.5% (Shalak et al. 2005 [55]) 0.3% (Sahni et al. 2019 [44]) 2.75% (Sayyed et al. 2020 [42])
Sepsis or pneumonia	Any sepsis or pneumonia – 8.2%	Culture positive sepsis /pneumonia 12 % (Yoder et al. 1983 [6]) Presumed sepsis or pneumonia – 14.6% (Venkatesh et al. 2019 [47])

Maternal intrapartum fever has been associated with a higher frequency of fetal tachycardia [15, 16], intervention for non-reassuring electronic fetal monitoring [17], operative vaginal delivery [18–20], cesarean delivery [15, 18–21], neonatal depression [15, 19–23], neonatal encephalopathy [22–27], perinatal arterial ischemic stroke [27, 28, 32], neonatal seizures [22–24, 27, 29–33], and NICU admission [15, 16, 19–21].

It is possible that antipyretic administration to patients with intrapartum fever can reduce adverse obstetric and neonatal outcomes. However, well-conducted studies about this matter are sparse. A more recent nonrandomized study compared the maternal and neonatal outcomes among 54 patients with PIT ≥ 38°C with and without antipyretic treatment. This study did not identify any difference in the frequency of cesarean delivery, presence of meconium, the requirement for neonatal bag/mask ventilation, requirement for continuous positive pressure ventilation, and NICU admission [12].

We hypothesized that the early use of paracetamol might abort further spike of fever and may blunt the clinical picture of CA. We compared the use of paracetamol between the two groups. In group 1, 88.2% of mothers received paracetamol when the temperature rose to > 37.5 ^0^C. Among group 2, ninety five mothers had documented temperature between 37.6 to 37.9^0^C before it increased to 38^0^C or more. However, only 38.9% of these mothers in group 2 received paracetamol for such mild elevation in temperature. This difference between groups 1 and 2 was statistically significant (*p*<0.001, OR 11.69, 95% CI 6.4 to 21.1) (Table 4). Our search did not identify published studies that examined the effect of prior use of paracetamol on the clinical picture of CA. Lesson T et al. [57]^.^ observed that in febrile parturients, paracetamol halted an increasing trend and stabilized the fetal temperature. In a double-blind placebo-controlled study, Goetzl et al. observed that prophylactic paracetamol did not prevent epidural-induced fever in nulliparous women [58].

## Limitations of the study

In addition to being retrospective, we were limited by the availability of a confirmed diagnosis. As both placental histopathology and amniotic fluid culture have limited utility in immediate patient care, these were not routinely performed in our practice. Placental histopathology was performed only among a small proportion of the study sample. Because of the limited utility and invasive nature, amniotic fluid aspiration was not performed in any of the subjects. We used placental culture as a surrogate for amniotic fluid culture.

## Conclusion

Our study observed that nearly half of the mothers with suspected clinical CA had PIT < 38^0^C. The overall maternal risk factors, maternal clinical and laboratory findings, and neonatal outcomes were similar to those in the previously published studies. There was no significant difference in the above factors or the rate of confirmed diagnosis between the low and higher temperature groups. We suspect that the use of antipyretics for intrapartum temperature <38^0^C may blunt the clinical picture of CA. Well-designed randomized prospective studies are required to examine the effect of antipyretics on clinical manifestations of CA. 

## Supplementary Information

**Additional file 1.** 

**Additional file 2.** 

**Additional file 3.**

## Data Availability

The datasets used and analyzed during the current study are uploaded as supporting documents.

## Abbreviations

AWH: Al Wakrah Hospital
CA: Chorioamnionitis
CRP: C-Reactive Protein
CSF: Cerebrospinal Fluid
LOCAL: Early Onset Sepsis Risk Calculator
EOS: Early-onset sepsis
GBS: Group B Streptococcus
IAI: Intra amniotic infection
MSAF: Meconium-Stained Amniotic Fluid
NICU: Neonatal Intensive Care Unit
PIT: Peak Intrapartum temperature
ROM: Rupture of membranes
SROM: Spontaneous rupture of membranes
WBC: White Blood Cells
