# Supplementary material for: Suspected clinical chorioamnionitis with peak intrapartum temperature <380C: the prevalence of confirmed chorioamnionitis and short term neonatal outcome
Source: BMC Pediatr. 2022 Apr 11;22:197. doi: 10.1186/s12887-022-03239-9 (PMC8996607; doi:10.1186/s12887-022-03239-9)
Supplement: Supplementary file 2 — Additional file 2. [file 12887_2022_3239_MOESM2_ESM.pdf]

مؤسسة حمد الطبية
Hamad Medical Corporation
HEALTH • EDUCATION • RESEARCH صحة • تعليم • بحوث

APPROVAL LETTER
MEDICAL RESEARCH CENTER
HMC, DOHA-QATAR

**Dr. Anvar Paraparambil Vellamgot**<br>**031309**<br>**Associate Consultant**<br>**Critical Care**<br>**Al Wakra Hospital (AWH)**<br>**Hamad Medical Corporation**	**Date: 30th August 2020**
**Protocol No.**	MRC-01-20-035
**Study Title**	Suspected chorioamnionitis with temperature <38degree Celcius -Prevalence of confirmed chorioamnionitis and short term neonatal outcome.
The above titled research study has been approved to be conducted in HMC summarized as below:	
**Study type:**	Data Review
**Data Collection Period:**	01/01/2016 to 31/12/2019
**Team Member List:**	Dr. Anvar Paraparambil Vellamgot , Dr. Hakam Khatib , Dr. Khalil Mohd. Khalil Salameh , Dr. Lina Hussain M. Habboub , Dr. Naser Abulgasim Mohamed Elkabir , Dr. Rajesh Pattu Valappil , Dr. Yousra Shahada Theeb Siam , Ms. Minu George
**Review Type :**	'Exempt' under MOPH guidelines<br>Category 3: Research involving the collection or study of existing: Data, documents, records and the information is recorded by the investigator in such a manner that subjects cannot be identified, directly or through identifiers linked to the subjects.
**Decision :**	Approved
**Hospitals/ Facilities Approved:**	Al Wakra Hospital (AWH)

This study must be conducted in full compliance with all the relevant sections of the Rules and Regulations for Research at HMC and the Medical Research Center should be notified immediately of any proposed changes to the study protocol that may affect the `exempt` status of this study. Wherever amendments to the initial protocol are deemed necessary, it is the responsibility of the Principal Investigator to ensure that appropriate reviews and renewed approvals are in place before the study will be allowed to proceed.

Please note that only research documentation uploaded in ABHATH currently are to be utilized at any stage in the conduct of this study. The research team must ensure that changes and progress on the study is appropriately recorded in ABHATH, the online research system of the Medical Research Center. PI must ensure that any link to patient identifiers must be destroyed after data collection and data security must to be maintained.

We wish you success in this research and await the outcomes in due course.

Yours sincerely,

Prof. Michael Paul Frenneaux
Chief of Scientific, Academic and Faculty Affairs
Hamad Medical Corporationn

Medical Research Centre
Hamad Medical Corporation - Doha - Qatar
حمد
Hamad

Date: 30th August 2020
